# The effects of RT-qPCR standards on reproducibility and comparability in monitoring SARS-CoV-2 levels in wastewater

**DOI:** 10.1038/s41598-024-77155-6

**Published:** 2024-10-26

**Authors:** Aapo Juutinen, Ananda Tiwari, Anna-Maria Hokajärvi, Oskari Luomala, Aleksi Kolehmainen, Eveliina Nurmi, Elisa Salmivirta, Tarja Pitkänen, Anssi Lipponen

**Affiliations:** 1 Department of Public Health, The Welfare Epidemiology and Monitoring Unit, Finnish Institute for Health and Welfare, Mannerheimintie 166, Helsinki, 00271 Finland; 2 Research Unit of Population Health, Faculty of Medicine, University of Oulu, Oulu, Finland; 3 Department of Public Health, Microbiology Unit, Finnish Institute for Health and Welfare, Kuopio, Finland; 4 Department of Food Hygiene and Environmental Health, Faculty of Veterinary Medicine, University of Helsinki, Helsinki, Finland; 5 Department of Medicine, Unit of Biomedicine, University of Eastern Finland, Kuopio, Finland

**Keywords:** Standard control, SARS-CoV-2, Wastewater surveillance, Wastewater-based epidemiology, Real-time quantitative PCR, Microbiology, Infectious diseases

## Abstract

Reverse transcription-quantitative PCR (RT-qPCR) is widely used for monitoring viruses, including severe acute respiratory syndrome coronavirus 2 (SARS-CoV-2), in wastewater. Various materials, including plasmid DNA, synthetic nucleic acids, PCR amplicons, genomic DNA, and cDNA, are currently used for SARS-CoV-2 quantification by generating standard curves. We assessed three common standards on quantifying SARS-CoV-2 RNA across nine wastewater treatment plants in Finland, as part of the national wastewater surveillance effort. We pairwise compared RT-qPCR results from 148 wastewater samples, using both IDT (#10006625, IDT, USA) and CODEX standards (#SC2-RNAC-1100, CODEX DNA), and 179 samples using both IDT and EURM019 standards (#EURM-019, European Commission, Joint Research Centre) in our assessment. Amongst the tested standards, the CODEX standard consistently yielded more stable results than either the IDT or EURM019 standards. We found that SARS-CoV-2 levels were higher with the IDT standard (4.36 Log_10_ GC/100 mL) compared to the CODEX standard (4.05 Log_10_ GC/100 mL). Similarly, quantification using the IDT standard was higher (5.27 Log_10_ GC/100 mL) than values obtained with the EURM019 (4.81 Log_10_ GC/100 mL). SARS-CoV-2 RNA quantified with IDT and CODEX standards exhibited stronger concordance (Spearman’s correlation rho median of 0.79) compared to those quantified with IDT and EURM019 standards (rho median of 0.59). This study highlights the significant impact of standard material selection on SARS-CoV-2 RNA quantification, emphasizing the need for harmonization in standard material.

**Supplementary Information:**

The online version contains supplementary material available at 10.1038/s41598-024-77155-6.

## Introduction

Wastewater surveillance is a widely employed method for monitoring the nucleic acid genomes of severe acute respiratory syndrome coronavirus 2 (SARS-CoV-2), as well as other pathogens, at the population level, complementing clinical level surveillance on individual cases^1–5^. It offers real-time information on outbreaks or changes in trends of virus loads in a given area in a resource-efficient manner, allowing the analysis of a large population with a relatively small number of samples^6–8^. Furthermore, wastewater surveillance can detect both symptomatic and asymptomatic cases without being influenced by any individuals’ test-seeking behavior, healthcare resources, or local testing strategy^6–9^. Typically, conventional clinical surveillance primarily focuses on symptomatic cases that require medical care, missing asymptomatic infections entirely. As a result, wastewater surveillance has been recognized as a reliable community-wide surveillance tool for monitoring COVID-19 trends and prevalence worldwide^1–3,8,10–12^.

Reverse transcription-quantitative PCR (RT-qPCR) is an ideal method for use in monitoring SARS-CoV-2 RNA levels in wastewater, combining high sensitivity (the ability to detect small amounts via amplification) and specificity (via the use of unique DNA sequences to the target)^1–4,13^. In order to quantify the amount of material in a sample, typically, a known standard material, bearing target nucleic acid specific sequences (e.g. plasmid DNA, synthetic nucleic acid, PCR amplicons, genomic DNA, or cDNA) is serially diluted 5 or 6 times (with steps of either 5 x or 10 x fold). Then each dilution is used in PCR, in order to amplify the target and the values are plotted together to create a standard or calibration curve, with the two axis being the known value of targets and the calculated instrument read values (typically fluorescence). If both the serial dilution of the standard and sample nucleic acids are amplified in the same PCR run, quantification cycle (Cq) (where the test values intersect the calibration curve) for test samples are compared to standard curve values to define the copy number of target nucleic acid in the sample^14,15^. Such use of quantified standard material enables results sharing and comparison among qPCR and RT-qPCR studies, allowing for consistent evaluation of gene copies per unit sample volume^4,16–18^.

Many factors can influence the quantification of SARS-CoV-2 RNA viral gene copies in samples. These can include the method of RNA extraction, RT-qPCR reagent types, sample quality and PCR platforms, as well as quantification approaches and the data analysis software employed^17,19–21^. This variability poses challenges for data sharing and comparison within and across laboratories^22^. Earlier studies have also indicated variations in SARS-CoV-2 RNA copies between different viral concentration methods^22–26^, quantification approaches (for example, dPCR or qPCR)^21,27^, as well as the selected primers, probes, and assays^3,28^. Variations in detected viral gene copy number are also common between the types of standard material such as plasmid DNA, synthetic DNA or RNA, PCR amplicons, genomic DNA, and cDNA used for generating the standard calibration curve on which sample quantification is based^4,14,16,18^.

Founded on our experience in continuous wastewater surveillance of SARS-CoV-2 (over three years), we have occasionally observed a rapid fluctuation in detected virus copy number between weekly samples, even when utilizing the same qPCR platform, virus concentration- nucleic acid extraction methods, and specific standard material used^3,28–30^. Few earlier wastewater studies suspected the possible effect of standard materials on the quantification of targets, but to our knowledge, not one has yet evaluated such effects^18,31^.

In this study, we compared three commonly used SARS-CoV-2 standard materials, a plasmid standard from IDT and two synthetic RNA standards, one from CODEX, and another one from the Joint Research Center (JRC) of the European Commission (EC), in a N2 RT-qPCR assay to quantify RNA gene copy numbers from wastewater^32^. We evaluated the variation of detected SARS-CoV-2 gene copy numbers and standard quality parameters (efficiency and Y-intercept) when using these standards. The findings of this study are intended to inform wastewater surveillance laboratories worldwide of the need to reduce variation in trend monitoring due to the selection of standard material and facilitate more reliable comparisons of results between laboratories in the field of wastewater surveillance for infectious diseases.

## Materials and methods

### Description and handling of standard materials

This study compared three standards for SARS-COV-2 RT-qPCR: (a) the plasmid standard from IDT (#10006625, 2019_nCoV_N Positive Control, IDT, USA, hereafter referred to as IDT), (b) the synthetic RNA standard from CODEX (#SC2-RNAC-1100, CODEX DNA (now TelesisBio) San Diego, CA, USA, (hereafter called CODEX)), and (c) the single-stranded RNA-standard from JRC (#EURM-019, European Commission, Joint Research Centre, Belgium, hereafter called EURM019) (Table 1). The standard curves were generated in each run of the RT-qPCR by using serial dilutions of the standard materials (Table 1). The IDT plasmid standard was used without linearization as previously described^18,28,33^.

**Table 1:** Properties and handling of standard material during this study.

	IDT	CODEX	EURM019
Properties			
DNA or RNA	Plasmid DNA	Synthetic RNA	Synthetic single stranded RNA
Stock concentration	2 × 10^5^ copies/µl	1 × 10^6^ copies/µl	1 × 10^8^ copies/µl
Recommend storage conditions	-15 to -30 °C	-80 °C	Original stock -20 °C, diluted aliquots -70°C or below
Cat. nro	10006625	SC2-RNAC-1100	EURM-019
Handling			
Stock concentration, storage, and handling	2 × 10^5^ copies/µl stock was divided to aliquots from which a dilution series was prepared for RT-qPCR. Dilution series was thawed 1–2 times per week and used maximum for one month. Stock and dilution series was kept in -20°C.	1 × 10^5^ copies/µl which was kept at -75 °C or below and thawed maximum for two times to prepare dilution series for RT-qPCR.	1 × 10^6^ copies/µl which was kept at -75°C or below and discarded after dilution series preparation for RT-qPCR.
Standard dilutions in RT-qPCR (GC/rxn)	5 × 10^4^, 5 × 10^3^, 5 × 10^2^, 5 × 10^1^, 5 × 10^0^	5 × 10^4^, 5 × 10^3^, 5 × 10^2^, 5 × 10^1^, 2.5 × 10^1^, 5 × 10^0^	5 × 10^4^, 5 × 10^3^, 5 × 10^2^, 5 × 10^1^, 1 × 10^1^, 5 × 10^0^

Various factors, including variable reaction conditions, instrument variations, preparation, handling, storage of the standard, as well as the freezing and thawing cycles during the laboratory working scheme, could influence the performance of standards^14,18–20,34,35^. To minimize these factors, RT-qPCR reactions for two standards for pairwise comparison was pipetted by same trained person, same qPCR thermocyclers and facilities were used, pipettes and qPCR cyclers were calibrated regularly, minimum volume of pipetting was 5 µl, same lot of standards were used during the experiments, cycling conditions, and master mix (see 2.4) for all three standards, maintaining the recommended storage conditions specified by each standard’s manufacturer (Table 1).

### Sample collection

A total of 327 twenty-four-hour composite influent samples (wastewater before treatment) were collected from nine wastewater treatment plants (WWTPs) in Finland (Table S1), between 8th June 2021 and 7th November 2022, as described earlier^28^. These WWTPs collectively serve about 2.4 million people, corresponding to 42.6% of the total population of Finland. The estimated population coverage of each of the WWTPs varied from 860 000 individuals in Viikinmäki, Helsinki to 69 500 individuals in Pått, Vaasa. One liter of the samples, from each WWTP, was transported in cool boxes within 24 h to the Water Microbiology Laboratory of the Finnish Institute for Health and Welfare (THL) located in Kuopio, Finland, for analysis^3,28^. A total of 148 pairwise measurements, with samples derived from the 8th June to the 13th December 2021, were carried out with the IDT and CODEX standards. With the IDT and EURM019 standards, 179 pairwise measurements, with samples taken from 27th June to the 7th November 2022, were performed.

### Nucleic acid extraction

The analysis of wastewater samples and nucleic acid extraction was done as described earlier^3,28^. Before extraction, mengovirus strain VMC0 (ATCC VR-1597, CeeramTOOLS, France) was spiked into the wastewater samples to act as an internal process control to estimate the recovery efficiency and RT-qPCR inhibition^3,28^. For ultrafiltration, 10 kDa Centricon Plus-70 centrifugal filters (#UCF7011008, Merck Millipore Ltd. Burlington, MA, USA) were used for 70 ml pre-centrifugated supernatants with a concentration-time of 25 min at 3000×*g* producing 200 µl–1600 µl of final concentrate. Total nucleic acid was extracted by using Chemagic Viral300 DNA/RNA extraction kit (#CMG-1033-S, PerkinElmer, Waltham, MA, USA) in conjunction with a Chemagic 360 instrument (PerkinElmer). Sterile deionized water was used as negative process control.

### RT-qPCR amplification and nucleic acid quantification

RT-qPCR assays to quantify SARS-CoV-2 and mengovirus were performed, as described earlier^3,28^. In brief, for SARS-CoV-2, primers 2019-nCoV_N2-F and 2019-nCoV_N2-R together with probe 2019-nCoV_N2-P (N2 assay)^32^ targeting the nucleocapsid protein gene was used, whilst for mengovirus specific sequences, primers mengo 110 and mengo 209, together with probe mengo 147 was used (Table S2)^32,36^. The RT-qPCR reactions and the target quantitation were carried out as previously described by Hokajärvi and colleagues^28^ using the TaqMan Fast Virus 1-step Master Mix (#4444436, Applied Biosystems, ThermoFisher Scientific). The same RT-qPCR cycling conditions were used for all three standards (Table S3). All standard dilutions points were analyzed in duplicate wells and samples in triplicate PCR reactions in 96-wellplate (see Table 1 for standard points). RT-qPCR inhibition of samples was evaluated by using a dilution method were non-diluted extracted nucleic acid and a 10-fold dilution of the nucleic acid was used as a template. Possible inhibition was followed by comparing mengovirus gene copy number in the samples to corresponding mengovirus copy number in negative process control analysed, without sample material. Molecular biology grade water (#BP2819100, Fisher BioReagents, Thermo Fisher Scientific, Pennsylvania, USA) was used for dilutions and as a non-template control during laboratory processing and the RT-qPCR. All PCRs were performed by using a QuantStudio 6 Flex real-time PCR system (Applied Biosystems, ThermoFisher Scientific, Pennsylvania, USA). N2 gene copies per 100 ml of wastewater were calculated for samples which had gene copies above of limit of quantification (LOQ) (see results 3.1). Gene copies per 100 ml of wastewater were calculated by dividing gene copy number per reaction with analyzed sample volume (including effective volume in every processing step) to get result as gene copies per ml and finally multiply that with 100.

### Statistical analysis

To compare variability in SARS-CoV-2 copy numbers produced with different materials to produce standard curves, the coefficient of variation (CV) was calculated with the equation:

1 $$\:CV\:=\:\frac{\sigma\:}{\mu\:}$$

Where CV = Coefficient of variation, $\:\sigma\:$ = standard deviation, and $\:\mu\:$ = mean.

A comparison of CVs rather than a comparison of variances was carried out since there were considerable differences in the magnitude of the virus-RNA copy numbers produced with the three standards from wastewater samples. The asymptotic test introduced by Feltz and Miller was used in the comparison of the CVs^37^ and a p-value of 0.05 was considered as a limit of statistical significance. A Wilcoxon signed-rank test^38^ was used to compare the actual (Log_10_) RNA copy numbers between the standards.

The relationship of SARS CoV-2 copy-numbers produced between the standard pairs (between IDT and CODEX, as well as between IDT and EURM019) was measured by using the Spearman’s correlation coefficient with 10 000 bootstrap replications. We used bias-corrected and accelerated bootstrap intervals (BCa) for the confidence intervals to deal with the potential skewness and bias in the bootstrap distribution^39^. Additionally, we employed a one-way analysis of variance (ANOVA) to scrutinize variations in both efficiency and slope means when comparing the two standard pairs (IDT against CODEX, and IDT against EURM019).

All statistical analysis was performed by RStudio (R version 4.2.2)^40^. R package *coequality*^41^, was used in the comparison of CVs, and *boot* package was used in the bootstrapping of the correlation coefficients^42^.

## Results

### Viral RNA extraction efficiency and RT-qPCR performances

To compare performance of IDT, EURM019 and CODEX standards in N2 assay, Y-intercept and slope of standard curves were compared.The median value of Y-intercept for standard curves generated in different batches of the experiment with the IDT plasmid standard was 40.07 (ranging from 38.7 to 41.39), whilst for the CODEX RNA standard, it was 39.55 (range 39.24 to 40.88). In the other standard’s comparison, the median Y-intercept value for the IDT plasmid was 39.9 (range 38.13 to 42.01) contrasting with the EURM019 RNA standard at 39.01 (range 36.79 to 41.1) (Table 2).

The median slope value of the IDT plasmid standard was -2.99 (range -3.36 to -2.79), whilst for the CODEX RNA standard it was -3.41 (range -3.66 to -3.28). In the subsequent comparison, the median slope for the IDT standard was -3.02 (ranging from -3.4 to -2.56) contrasting with that obtained with the EURM019 RNA standard at -3.27 (range -3.67 to -2.98) (Table 1) with N2 assay. The slopes between IDT and CODEX standard pair, and between the IDT and CODEX standard pair differed significantly (ANOVA *p* < 0.001) (Tables S4 and S5).

For all three standards limit of detection (LOD) was Cq 40 as described earlier in Tiwari et al.^3^. LOQ for IDT was 50, CODEX 25 and EURM019 5 gene copies/reaction.

**Table 2:** Performance characteristics of the RT-qPCR assays. Number of RT-qPCR runs (batches of experiments; n).

Assays		Y-intercept	Efficiency (%)	Slope	Correlation coefficient
IDT (*n* = 24)	Minimum	38.7	98.5 1	-3.36	0.98
	Maximum	41.39	128.47	-2.79	1.00
	Median	40.07	115.75	-2.99	0.99
	Average	39.97	116.1	-3.0	0.99
CODEX (*n* = 24)	Minimum	39.24	87.61	-3.66	0.99
	Maximum	40.88	101.91	-3.28	1.00
	Median	39.55	96.53	-3.41	1.00
	Average	39.77	96.04	-3.42	0.99
IDT (*n* = 28)	Minimum	38.13	96.65	-3.4	0.95
	Maximum	42.01	145.9	-2.56	1.00
	Median	39.9	114.29	-3.02	0.99
	Average	40.09	115.68	-3.02	0.99
EURM019 (*n* = 28)	Minimum	36.79	87.37	-3.67	0.97
	Maximum	41.1	116.49	-2.98	1.00
	Median	39.01	102.37	-3.27	0.99
	Average	39.14	101.1	-3.31	0.99

The recovery efficiencies for the mengovirus with undiluted nucleic acid ranged from 0.9 to 250%, with a median of 19.5%, which is similar with our previous study^28^. Recovery above 1% indicates sufficient yield during laboratory analysis process^43^. In rare cases (*n* = 1) where mengovirus yield was below 1%, a 10-fold dilution was used for SARS-CoV-2 quantification to avoid inhibition.

### SARS-CoV-2 RNA copy number calculations and their variation between the different standard materials used

**Fig. 1:**
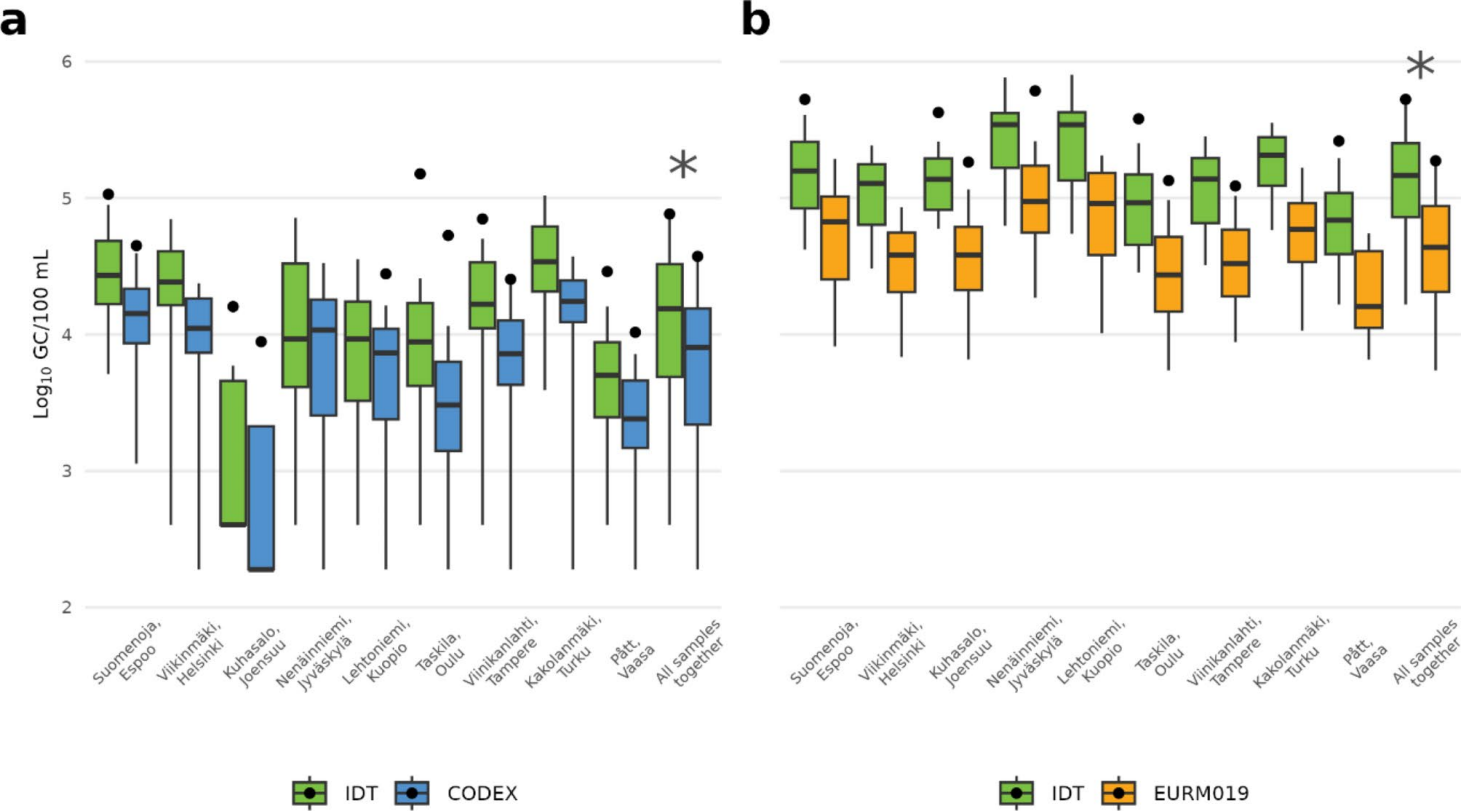
SARS-CoV-2 RNA copy numbers detected using the IDT plasmid, and CODEX and EURM019 RNA standards from wastewater (**a**) and using IDT plasmid standard and EURM019 RNA (**b**). Wastewater samples collected from nine wastewater treatment plants in Finland. The number of samples per WWTP ranged from 12 to 20 (see Table 3). The IDT standard consistently yielded significantly higher copy numbers than the CODEX standard in all samples together (*p < 0.001, Wilcoxon signed-rank test). Similarly, the IDT standard consistently yielded significantly higher virus copy numbers than EURM019 standard (*p* < 0.001, Wilcoxon signed-rank test). Since small sample number of individual WWTP, we did not carryover statistical test for them. IDT, CODEX and EURM019 indicated in light green and light blue and orange boxes, respectively.

To compare the effect of IDT, EURM019 and CODEX standards on wastewater results, we calculate gene copy numbers in wastewater by using standard curves generated by these standards. The Wilcoxon signed-rank test, a non-parametric test used to compare paired samples, was chosen due to its robustness in detecting differences between related samples without assuming a normal distribution. This test was particularly relevant given the potential variability and non-normal distribution of RNA copy numbers across different standards. When using IDT plasmid standard to produce SARS-CoV-2 copy numbers from wastewater samples, on average RNA copy numbers were significantly higher (4.36 Log_10_ GC/100 mL) than when using the CODEX RNA standard (4.05 Log_10_ GC/100 mL) (*p* < 0.001, Wilcoxon signed-rank test), when comparing samples from nine WWTPs together (Fig. 1a). Similarly, the SARS-CoV-2 RNA results (5.27 Log_10_ GC/100 mL) were significantly higher when the IDT standard was used, in comparison with the EURM019 RNA standard (4.81 Log_10_ GC/100 mL) (*p* < 0.001, Wilcoxon signed-rank test) (Fig. 1b). This indicates that the IDT standard tends to produce higher RNA copy numbers over CODEX and EURM019 standards.

Amongst the three tested RT-qPCR standard materials, CODEX had the lowest variance in SARS-CoV-2 RNA copy number when data from the nine WWTPs together. The variance (σ^2^) of the IDT plasmid standard (8.79 Log_10_ GC/100 mL) was higher than CODEX RNA standard (8.29 Log_10_ GC/100 mL) (Supplementary Fig. 1). In addition, the σ^2^ of the IDT standard was higher (10.4 Log_10_ GC/100 mL) than the EURM019 standard (9.64 Log_10_ GC/100 mL) (Supplementary Fig. 2). This indicates that the CODEX standard has higher reproducibility and less variation when compared to the IDT and EURM019 standards.

When we compared the coefficients of variation (CV) in the SARS-CoV-2 copy number produced with the three different standards, no significant difference was observed between the CV of IDT (1.1) and CODEX (1.2) (p-value: 0.4, Feltz and Miller asymptotic test). The Feltz and Miller asymptotic test is specifically designed to compare CVs between independent groups, making it particularly useful for assessing the variability of RNA copy number measurements across different standards. In contrast, the statistical analysis revealed a significant difference between the CV of IDT (0.8) and EURM019 (1.0) (p-value: 0.03, Feltz and Miller asymptotic test). This finding indicates the potential impact of standard selection on the reliability and reproducibility of SARS-CoV-2 RNA measurements in wastewater samples.

### Correlation between SARS-CoV-2 copy numbers produced with the three tested standard materials

To test correlation of wastewater gene copy numbers produced with standards, the pairwise correlation coefficients between the gene copy numbers produced with the CODEX and EURM019 standards was compared with those obtained from using the IDT plasmid standard using Spearman’s correlation coefficients (95% BCa confidence intervals based on 10 000 bootstrap replications) (Table 3). The number of pairwise observations was stable (ranging from 19 to 20) between IDT and EURM019 standards pairs among samples analyzed from the WWTPs. When comparing SARS-CoV-2 copy numbers produced with the IDT and CODEX standards the number of pairwise observations ranged from 12 to 20 between WWTP’s due to differences in the numbers of available RT-qPCR results. While calculating the Spearman’s correlation between IDT and EURM019 standards none of the WWTP-specific datasets out of nine exceed S_r_ values more than 0.80 (min 0.45, max 0.77, median = 0.59). When the correlation between IDT and CODEX standards was analyzed, the correlation coefficient with samples from four out of the nine WWTPs exceeded the S_r_ value of 0.8 (min 0.45, max = 0.88, median = 0.79).

**Table 3:** The number of pairwise observations, Spearman correlation coefficients (rho) and confidence intervals between the SARS-CoV-2 copy numbers produced with IDT plasmid and EURM019 RNA, and between IDT and CODEX standards on samples from the nine WWTPs.

WWTP, location	IDT versus CODEX			IDT versus EURM019		
	Number of paired RT-qPCR results	Spearman´s rho (Sr)	95% BCa confidence Interval	Number of paired RT-qPCR results	Spearman´s rho (Sr)	95% BCa confidence Interval
Suomenoja, Espoo	18	0.45	-0.01, 0.8	20	0.51	0.01, 0.82
Viikinmäki, Helsinki	19	0.77	0.35, 0.93	20	0.45	-0.01, 0.77
Kuhasalo, Joensuu	13	0.88	0.54, 1.0	20	0.59	0.17, 0.83
Nenäinniemi, Jyväskylä	19	0.79	0.4, 0.94	20	0.57	0.01, 0.85
Lehtoniemi, Kuopio	12	0.73	-0.04, 0.98	20	0.77	0.44, 0.93
Taskila, Oulu	15	0.66	-0.2, 0.96	20	0.65	0.09, 0.89
Viinikanlahti, Tampere	20	0.82	0.47, 0.94	20	0.65	-0.02, 0.89
Kakolanmäki, Turku	18	0.85	0.64, 0.96	20	0.56	0.07, 0.84
Pått, Vaasa	14	0.88	0.56, 0.98	19	0.59	-0.07, 0.88
Median Sr of nine WWTPs		0.79			0.59	
Altogether, (95% BCa Confidence Interval of Sr)	**0.83 (0.74**,** 0.89)**			**0.69 (0.58**,** 0.78)**		

*BCa* bootstrap intervals, *WWTP* wastewater treatment plant

## Discussion

This study compared the quantification and variability of SARS-CoV-2 RNA gene copies measured from wastewater samples when using a variety of different standards (a plasmid DNA standard from IDT, a synthetic RNA standard from CODEX and a synthetic RNA standard from European Commission Joint Research Centre (EURM019)) for generating RT-qPCR standard curves in an N2 assay. Discrepancies were found in the pairwise comparisons of SARS-CoV-2 RNA copy numbers and variation when analyzing wastewater samples with these standards. Amongst the tested standards SARS-CoV-2 analysis with the N2 RT-qPCR assay, the CODEX RNA standard produced consistently more stable results when compared to both the IDT plasmid and EURM019 RNA standards. We also found that the standard tested tended to have differences in slope and efficiency in RT-qPCR. However, strong correlations in RNA copy numbers were noted between different standards, whilst varying between samples derived from different WWTPs.

When we tested the variation and level in detected gene copy numbers in wastewater using the IDT, CODEX and EURM019 standards, we found that IDT produced higher copy numbers than CODEX and EURM019 standard. Several factors might contribute to the differences found in detected copy numbers when employing different standard materials in the N2 RT-qPCR assay. Among the three tested standards, there was a variation in the concentration of the supplied stocks between manufacturers and some differences in handling. In addition, the characteristics of the three standards were different, as IDT was plasmid DNA whereas EURM019 and CODEX were synthetic RNA. Using plasmid DNA standards for quantifying RNA targets leads to one-step RT-qPCR omitting cDNA synthesis and so leaving one part of the assay uncontrolled which is not optimal for quantification. This suggests that the impact of using diverse standard materials on RT-qPCR amplification performance is frequently overlooked in wastewater surveillance which was also pointed out in review by Bivins and colleagues^18^.

In addition to differences in variation between standards, we also observed differences in slope and efficiency in generated standard curves between standards. The ideal slope of the standard curve is -3.32 which indicates 100% RT-qPCR amplification efficiency, although a range from  -3.1 (110%) to -3.58 (90%) is a typically accepted efficiency values for an optimized probe-based assay^17^. The efficiency of synthetic RNA-based CODEX and EURM019 standards was within acceptable limits (90–105%)^17^. However, in more than half of the RT-qPCR runs, the amplification efficiency of the IDT standard exceeded 115%, and was considered higher than anticipated according to Minimum Information for Publication of Quantitative Real-time PCR (MIQE) guidelines^17^. This could be due to the circularity of the plasmid or stock handling which differs from EURM019 and CODEX standards. Existing literature on the effect between circular and linear plasmids on efficiency presents contradictory findings, with some studies indicating a significant effect and others showing no impact^18,34^. A review by Bivins and colleagues^18^, focusing on the available wastewater surveillance literature for SARS-CoV-2, highlighted similar effects of standard materials on slope and efficiency as in our study^18^. Several other factors, such as variable reaction conditions, errors in standard dilution preparation, instrument variations, preparation, handling, storage of the standard, its storage time after opening in the laboratory, as well as freezing and thawing cycles during batches of experiments, could also influence these efficiencies^14,18,34^. We minimized these factors by pipetting PCR reactions by trained personnel, calibrating pipettes regularly, using same facilities, qPCR thermocycler, cycling program and master mix and following manufacturers recommend storage conditions for stocks.

Given the differences in detected gene copy numbers and PCR efficiency among the standards, we tested the correlation of gene copy numbers in wastewater produced by all three standards. We found a strong correlation in the copy numbers produced using IDT, CODEX, and EURM019 standards. This suggests that, over the long term, the trends between the standards are similar, even though they tend to produce different levels of gene copy numbers. However, since IDT exhibited the most variation in efficiency, the gene copy numbers produced by IDT might show more variation in the short term. Among the tested standards, the IDT was reported to be the most commonly used in wastewater surveillance of SARS-CoV-2 assays^18^. An earlier study reported that plasmids were used in 28.4% of studies, whilst synthetic RNA at 17.3%, synthetic DNA at 13.9%, various forms of transcripts at 9.1% as well as 31.3% were not or improperly reported for quantification of SARS-CoV-2 in wastewater surveillance out of total 208 assays in their review of 81 publications^18^. Reporting of RT-qPCR results with adequate information about the PCR technologies, reagents, protocols, analysis methods, and standard parameters, as recommended by the MIQE guidelines, is essential^17,18^. Currently, there is no consensus within wastewater surveillance regarding monitoring methods and standard materials for RT-qPCR and dPCR for monitoring SARS-CoV-2^4,14,21^. Establishing a consensus on methods and standards could enhance the reliability of the analysis and enable comparison of wastewater surveillance results across different laboratories over different timeframes and locations. Furthermore, international standardization of the wastewater surveillance methodology is key for enabling harmonized surveillance practices and comparison possibilities between different surveillance programs. Nevertheless, when it comes to inter-laboratory comparisons, using different standard materials may not be ideal since they tend to produce different relative copy number levels. The same problem might arise when the standard material or batch of the standard changes during long-term surveillance efforts. For this, Nagelkerke and colleagues introduced a method that combines standard-curve data in a rolling 5-day window, derived from four to ten daily PCR runs^44^. In the future, we intend to use a time-series statistical model to normalize variation of the standard efficiency between sampling timepoints, however this would require a much longer time series and larger sample numbers than we have at the moment.

As a limitation of our study, we did not conduct a parallel comparison between EURM019 and CODEX standards. However, we believe that this comparison of the most widely used IDT plasmid standard to two different RNA standards is useful. We paid careful attention to keeping all experimental conditions the same for all three standards and performed the RT-qPCR with two standards simultaneously in the same 96-well plate. The study herein covers about one year’s worth of data from a nationwide (Finland) COVID-19 wastewater surveillance, and RT-qPCR was carried out across multiple experimental batches and wastewater sampling sites.

## Conclusions

Our findings suggest using RNA-based standards instead of plasmid standards when measuring RNA targets, as they tend to exhibit optimal efficiency and smaller variation between RT-qPCR runs. While differences were observed in copy numbers, efficiency, and variation in SARS-CoV-2 results with different standards, we found a strong correlation in the data produced by all three standards, indicating their consistency in detecting trends in SARS-CoV-2 RNA amounts in wastewater. Additionally, RNA-based standards control for cDNA synthesis-based effects, which is not the case with DNA or plasmid standards. According to our knowledge no study has, to date, compared the impact of different RT-qPCR standard materials on SARS-CoV-2 monitoring in wastewater surveillance. As highlighted here, the selection of RT-qPCR standards can influence the quantification and variance of the measured SARS-CoV-2 RNA copy numbers in wastewater. This also indicates the importance of standard selection when measuring other pathogens from wastewater. In summary, our study i that RNA-based standards (CODEX and EURM019) exhibit less variability compared to the circular plasmid standard (IDT), and the using of RNA standards could simplify result interpretation for RNA targets during pandemic assessment phases.

## Electronic supplementary material

Below is the link to the electronic supplementary material.

Supplementary Material 1

## Data Availability

The data of IDT and EURM019 standards are available in https://www.thl.fi/episeuranta/jatevesi/wastewater_weekly_report.html by CC BY 4.0 license. Data of CODEX standard is available from the authors upon reasonable request.
