# Supplementary material for: The effects of RT-qPCR standards on reproducibility and comparability in monitoring SARS-CoV-2 levels in wastewater
Source: Sci Rep. 2024 Oct 26;14:25582. doi: 10.1038/s41598-024-77155-6 (PMC11513023; doi:10.1038/s41598-024-77155-6)
Supplement: Supplementary file 1 — Supplementary Material 1 [file 41598_2024_77155_MOESM1_ESM.docx]

**The effects of RT-qPCR standards on reproducibility and comparability in monitoring SARS-CoV-2 levels in wastewater**

Aapo Juutinen^a, b, #^, Ananda Tiwari^c, d, #^, Anna-Maria Hokajärvi^c^, Oskari Luomala^a^, Aleksi Kolehmainen^c^, Eveliina Nurmi^c^, Elisa Salmivirta^c^, Tarja Pitkänen^c,d^, Anssi Lipponen^c,e*^

^a^Department of Health Security, Finnish Institute for Health and Welfare, Mannerheimintie 166, 00271, Helsinki, Finland

^b^Research Unit of Population Health, Faculty of Medicine, University of Oulu, Finland

^c^Department of Health Security, Expert Microbiology Research Unit, Finnish Institute for Health and Welfare.

^d^Department of Food Hygiene and Environmental Health, Faculty of Veterinary Medicine, University of Helsinki, Finland.

^e^University of Eastern Finland, Department of Medicine, Unit of Biomedicine, Kuopio, Finland.

# AJ and AT contributed equally and shared the first authorship.

*Corresponding author: Anssi Lipponen ([anssi.lipponen@thl.fi](mailto:anssi.lipponen@thl.fi))

| **Supplemental table S1.** Description of wastewater treatment plants in this study^1^. | | | | | |
| --- | --- | --- | --- | --- | --- |
| **WWTP, location** | **Municipalities served by the WWTP** | **Number of samples included (N)** | **Population served / total in the area (coverage)** | **24h-composite sampling**  **method** | **Industrial wastewater proportion**  **%** |
| Viikinmäki, Helsinki | Helsinki, Sipoo, Kerava, Tuusula, Järvenpää, Pornainen, Mäntsälä, Vantaa (half of the population) | 39 | 860 000 / 946 321 (0.91) | Flow and time | 7 |
| Suomenoja, Espoo | Espoo, Vantaa (half of the population), Kirkkonummi, Kauniainen, Siuntio | 38 | 390 000 / 465 142 (0.84) | Flow | 5 |
| Kakolanmäki, Turku | Turku, Naantali, Masku, Raisio, Kaarina, Paimio, Mynämäki, Nousiainen, Rusko, Lieto, Aura, Pöytyä, Oripää, Marttila | 38 | 300 000 /346 367 (0.87) | Flow | 15 |
| Taskila, Oulu | Oulu, Muhos, Utajärvi, Ii | 35 | 200 000 / 227 297 (0.88) | Time | NA |
| Viinikanlahti, Tampere | Tampere, Kangasala, Pirkkala, Lempäälä | 40 | 200 000 /314 171 (0.64) | Flow | NA |
| Nenäinniemi, Jyväskylä | Jyväskylä, Laukaa, Muurame, Uurainen | 39 | 154 600 /175 415 (0.88) | Flow | 10 |
| Kuhasalo, Joensuu | Joensuu, Kontiolahti, Liperi, Polvijärvi | 33 | 98 000 /108 120 (0.91) | Flow | 20 |
| Lehtoniemi, Kuopio | Kuopio | 32 | 90 697 /119 533 (0.76) | Flow and time | NA |
| Pått, Vaasa | Vaasa, Mustasaari, Maalahti | 33 | 69 500 /92 576 (0.75) | Flow | NA |
| **Total** |  | **327** |  |  |  |

| **Supplemental table S2. Nucleic acid sequences of the primers and probes used in the study to detect SARS-CoV-2 and Mengovirus.** | | | |
| --- | --- | --- | --- |
| Target virus | Oligonucleotide | Sequence and label (5' → 3') | Reference |
| SARS-CoV-2 | 2019-nCoV_N2-F | TTACAAACATTGGCCGCAAA | Lu *et al.* 2020^2^ |
|  | 2019-nCoV_N2-R | GCGCGACATTCCGAAGAA | Lu *et al.* 2020^2^ |
|  | 2019-nCoV_N2- P | FAM-ACAATTTGCCCCCAGCGCTTCAG-BHQ1 | Lu *et al.* 2020^2^ |
| Mengovirus | Mengo 110 | GCG GGT CCT GCC GAA AGT | Pintó et al. 2009^3^ |
|  | Mengo 209 | GAA GTA ACA TAT AGA CAG ACG CAC AC | Pintó et al. 2009^3^ |
|  | Mengo 147 | FAM-ATC ACA TTA CTG GCC GAA GC-MGB | Pintó et al. 2009^3^ |

FAM: 6-carboxyfluorescein; BHQ1: Black Hole Quencher 1; MGB: Minor Groove Binder.For SARS-CoV-2 and Mengovrus primers were purchased from IDT with standard purification. SARS-CoV-2 probe were purchased from IDT and for Mengovirus from Applied Biosystems with HPLC purification.

**Supplemental Table S3. Details of RT-qPCR reactions and the cycling conditions used in the study.**

| Target | Reaction volume | RT-qPCR reaction mix | Thermal cycling conditions | Reference |
| --- | --- | --- | --- | --- |
| SARS-CoV-2 | 25 μl | RNA sample: 5 μl  Primers: 0.2 μM  Probe: 0.2 μM  TaqMan Fast Virus 1-step Master Mix: 6.25 ul | 50°C for 5 min for reverse transcription, followed by 95°C for 20 s, and then 45 cycles of 95°C for 15 s and 58°C for 1 min | Lu *et al.* 2020^2^,  Medema *et a*l. 2020^4^ |
|  |  |  |  |  |
|  |  |  |  |  |
|  |  |  |  |  |
| Mengo-virus | 25 μl | RNA sample: 5 μl  Primers: 0.8 μM  Probe: 0.2 μM  TaqMan Fast Virus 1-step Master Mix: 6.25 ul | 50°C for 5 min for reverse transcription, followed by 95°C for 20 s, and then 45 cycles of 95°C for 15 s and 60°C for 1 min. | Pintó et al. 2009^3^,  ISO/TS 15216-1:2017^5^ |
|  |  |  |  |  |
|  |  |  |  |  |
|  |  |  |  |  |

| **Supplemental table S4. Comparison of the slopes between the standards.** ANOVA indicated differences between IDT vs. CODEX and IDT vs. EURM019 (p<0.001). | | | | | | |  |
| --- | --- | --- | --- | --- | --- | --- | --- |
|  |  | Df | Sum.Sq | Mean.sq | F value | P-value | |
| IDT vs. CODEX | Between groups | 1 | 2.2 | 2.2 | 136.9 | < 0.001 | |
|  | Within groups | 46 | 0.7 | 0.02 |  |  | |
| IDT vs. EURM019 | Between groups | 1 | 1.2 | 1.2 | 29.1 | < 0.001 | |
|  | Within groups | 54 | 2.2 | 0.04 |  |  | |

| **Supplemental table S5. Comparison of the efficiencies between the standards.** ANOVA indicated differences between IDT vs. CODEX and IDT vs. EURM019 (p<0.001). | | | | | | |
| --- | --- | --- | --- | --- | --- | --- |
|  |  | Df | Sum.Sq | Mean.sq | F value | P-value |
| IDT vs. CODEX | Between groups | 1 | 4829 | 4829 | 122.8 | < 0.001 |
|  | Within groups | 46 | 1809 | 39 |  |  |
| IDT vs. EURM019 | Between groups | 1 | 2977 | 2977.2 | 28.58 | < 0.001 |
|  | Within groups | 54 | 5625 | 104.2 |  |  |


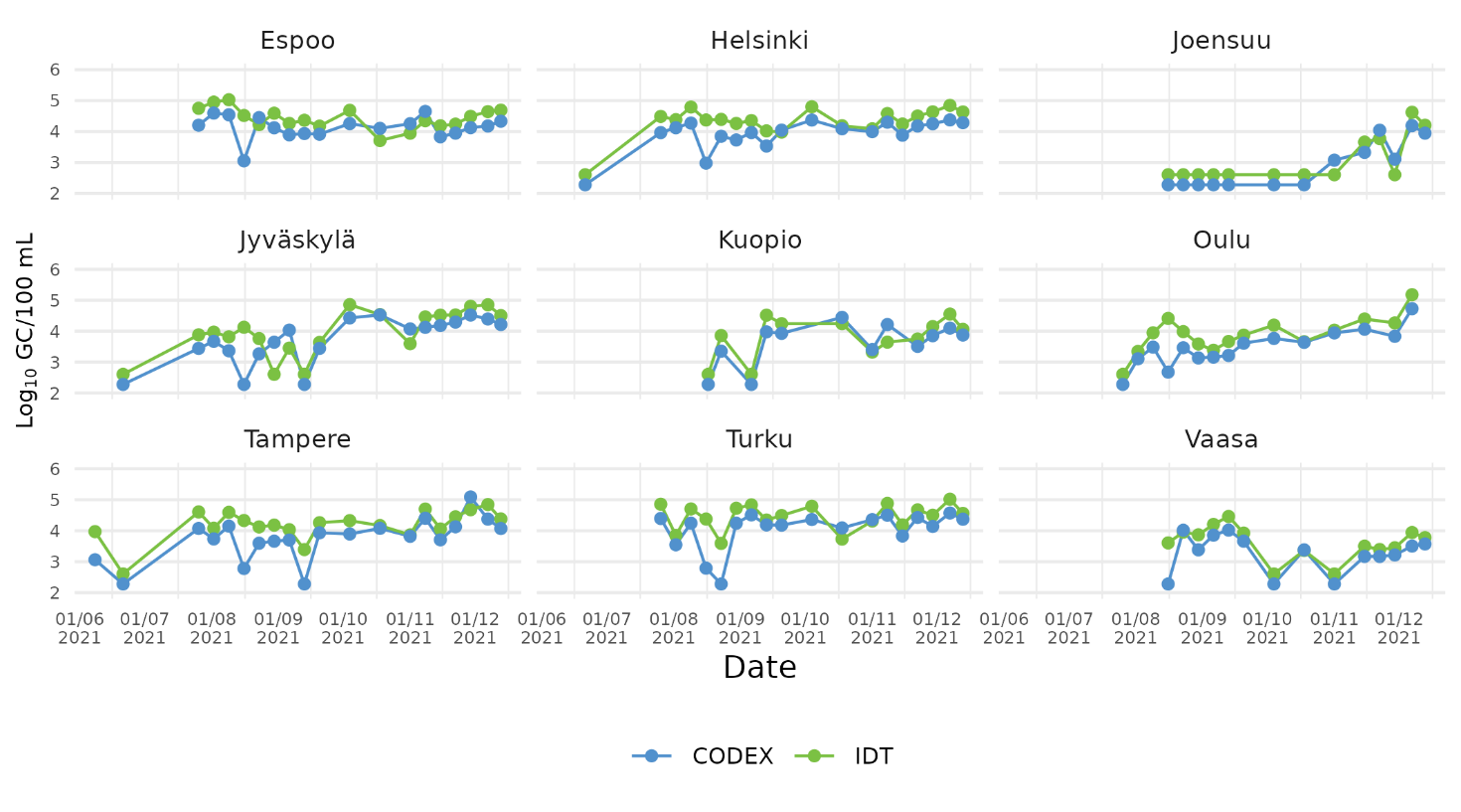
**Supplemental figure S1. Time series of number of virus RNA-copies with IDT and CODEX standards by municipality.** IDT samples in green, CODEX in blue.


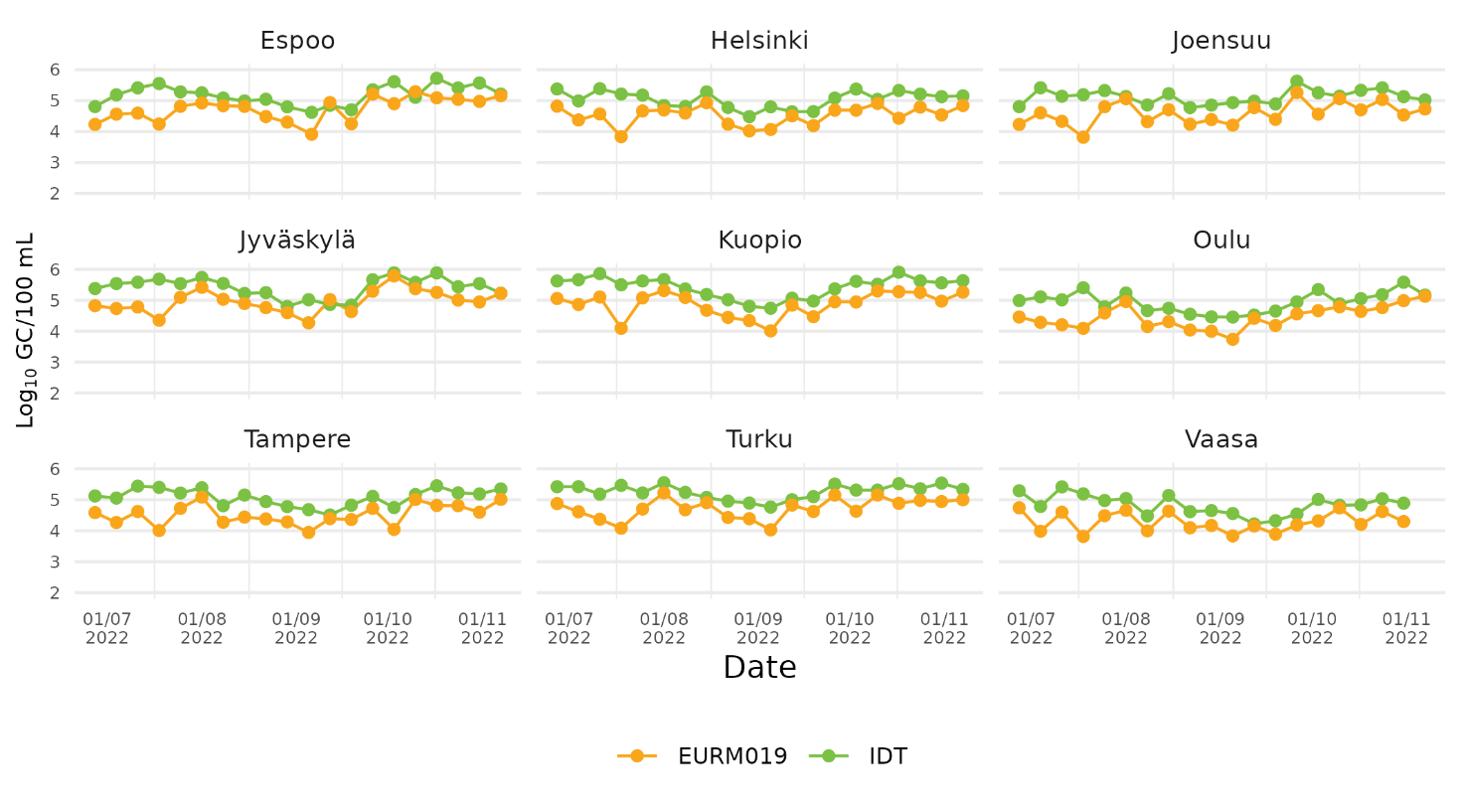
**Supplemental figure S2. Time series of number of virus RNA-copies with IDT and EURM019 standards by municipality.** IDT samples in green, EURM019 in orange.

MIQE checklist

| **ITEM TO CHECK** | **IMPORTANCE** | **CHECKLIST** |
| --- | --- | --- |
| **EXPERIMENTAL DESIGN** |  |  |
| Definition of experimental and control groups | **E** | Provided in materials and methods. |
| Number within each group | **E** | Provided in materials and methods. |
| Assay carried out by core lab or investigator's lab? | D | In laboratory of Finnish Institute for Health and Welfare laboratory, Kuopio, Finland. |
| Acknowledgement of authors' contributions | D | See authors' contributions in main text. |
| **SAMPLE** |  |  |
| Description | **E** | Total RNA extracted from wastewater. |
| Volume/mass of sample processed | D | See materials and methods section 2.3 for details. |
| Microdissection or macrodissection | **E** | Not relevant to this study |
| Processing procedure | **E** | See materials and methods section 2.3 for details. |
| If frozen - how and how quickly? | **E** | Not relevant to this study |
| If fixed - with what, how quickly? | **E** | Not relevant to this study |
| Sample storage conditions and duration (especially for FFPE samples) | **E** | Fresh wastewater samples were used. Standard by description in Materials and Methods |
| **NUCLEIC ACID EXTRACTION** |  |  |
| Procedure and/or instrumentation | **E** | See materials and methods section 2.3 |
| Name of kit and details of any modifications | **E** | See materials and methods section 2.3 |
| Source of additional reagents used | D | Not relevant to this study |
| Details of DNase or RNAse treatment | **E** | Not relevant to this study |
| Contamination assessment (DNA or RNA) | **E** | Not relevant to this study |
| Nucleic acid quantification | **E** | Not relevant to this study |
| Instrument and method | **E** | Not relevant to this study |
| Purity (A260/A280) | D | Not relevant to this study |
| Yield | D | Not relevant to this study |
| RNA integrity method/instrument | **E** | Not relevant to this study |
| RIN/RQI or Cq of 3' and 5' transcripts | **E** | Not relevant to this study |
| Electrophoresis traces | D | Not relevant to this study |
| Inhibition testing (Cq dilutions, spike or other) | **E** | See materials and methods section 2.4 |
| **REVERSE TRANSCRIPTION** |  |  |
| Complete reaction conditions | **E** | One step RT-qPCR mastermix were used. See materials and methods section 2.4 |
| Amount of RNA and reaction volume | **E** | 5µl of template nucleic acid in 25µl reaction |
| Priming oligonucleotide (if using GSP) and concentration | **E** | One step master mix used and so unknown |
| Reverse transcriptase and concentration | **E** | One step master mix used and so unknown |
| Temperature and time | **E** | 50C 5 min. See suppplementary Table S3. |
| Manufacturer of reagents and catalogue numbers | D | Applied Biosystems, ThermoFisher Scientific, #4444436, |
| Cqs with and without RT | D* | Not applicable for this study |
| Storage conditions of cDNA | D | Not applicable for this study |
| **qPCR TARGET INFORMATION** |  |  |
| If multiplex, efficiency and LOD of each assay. | **E** | Not applicable for this study |
| Sequence accession number | **E** | N2 gene of SARS-CoV-2 by 2019-nCoV_N2-F and 2019-nCoV_N2-R primers (Lu *et al*. 2020). |
| Location of amplicon | D | N2 gene |
| Amplicon length | **E** | 67 bp (Lu et al. 2020) |
| *In silico* specificity screen (BLAST, etc) | **E** | See (Lu et al. 2020) |
| Pseudogenes, retropseudogenes or other homologs? | D | See (Lu et al. 2020) |
| Sequence alignment | D | See (Lu et al. 2020) |
| Secondary structure analysis of amplicon | D | See (Lu et al. 2020) |
| Location of each primer by exon or intron (if applicable) | **E** | See (Lu et al. 2020) |
| What splice variants are targeted? | **E** | See (Lu et al. 2020) |
| **qPCR OLIGONUCLEOTIDES** |  |  |
| Primer sequences | **E** | Supplemental Table S2 |
| RTPrimerDB Identification Number | D | Not applicable for this study |
| Probe sequences | D** | Supplemental Table S2 |
| Location and identity of any modifications | **E** | No modifications were done |
| Manufacturer of oligonucleotides | D | IDT |
| Purification method | D | N2 Primers with standard purifiaction and probes with HPLC. |
| **qPCR PROTOCOL** |  |  |
| Complete reaction conditions | **E** | See materials and methods section 2.4 |
| Reaction volume and amount of cDNA/DNA | **E** | By one step mastermix (Applied Biosystems, ThermoFisher Scientific, #4444436), see supplemental Table S3. |
| Primer, (probe), Mg++ and dNTP concentrations | **E** | By one step mastermix (Applied Biosystems, ThermoFisher Scientific, #4444436) |
| Polymerase identity and concentration | **E** | By one step mastermix (Applied Biosystems, ThermoFisher Scientific, #4444436) |
| Buffer/kit identity and manufacturer | **E** | By one step mastermix (Applied Biosystems, ThermoFisher Scientific, #4444436) |
| Exact chemical constitution of the buffer | D | By one step mastermix (Applied Biosystems, ThermoFisher Scientific, #4444436) |
| Additives (SYBR Green I, DMSO, etc.) | **E** | Not used. |
| Manufacturer of plates/tubes and catalog number | D |  |
| Complete thermocycling parameters | **E** | Supplemental Table S3 |
| Reaction setup (manual/robotic) | D | Manual pipeting. |
| Manufacturer of qPCR instrument | **E** | QuantStudio 6 Flex real-time PCR system (Applied Biosystems, ThermoFisher Scientific) |
| **qPCR VALIDATION** |  |  |
| Evidence of optimisation (from gradients) | D |  |
| Specificity (gel, sequence, melt, or digest) | **E** | Supplemental table S2 and (Lu et al. 2020) |
| For SYBR Green I, Cq of the NTC | **E** | Not relevant to this study |
| Standard curves with slope and y-intercept | **E** | See results 3.1, Table 2 |
| PCR efficiency calculated from slope | **E** | See results 3.1, Table 2 |
| Confidence interval for PCR efficiency or standard error | D | See results 3.1, Table 2 |
| r2 of standard curve | **E** | See results 3.1, Table 2 |
| Linear dynamic range | **E** | See results 3.1 |
| Cq variation at lower limit | **E** | See results 3.1 |
| Confidence intervals throughout range | D |  |
| Evidence for limit of detection | **E** | See results 3.1 |
| If multiplex, efficiency and LOD of each assay. | **E** | Not applicable for this study |
| **DATA ANALYSIS** |  |  |
| qPCR analysis program (source, version) | **E** | QuantStudio Real-Time PCR Software v1.7.2 |
| Cq method determination | **E** | Threshold |
| Outlier identification and disposition | **E** | QuantStudion QC tools that uses calculated confidence in between replicates. If over 2 standarpoint outliers in standard curve, experiment was repeated. |
| Results of NTCs | **E** | NTCs did not exceeded threshold. |
| Justification of number and choice of reference genes | **E** | Not relevant to this study |
| Description of normalisation method | **E** | Not applicable for this study |
| Number and concordance of biological replicates | D |  |
| Number and stage (RT or qPCR) of technical replicates | **E** | 2 techincal relpicates in RT-qPCR for wastewater samples and each standard points. |
| Repeatability (intra-assay variation) | E | See results 3.1, Table 2 |
| Reproducibility (inter-assay variation, %CV) | D | Not applicable for this study |
| Power analysis | D | Not applicable for this study |
| Statistical methods for result significance | **E** | See materials and methods |
| Software (source, version) | E | QuantStudio 6 Flex real-time PCR system (Applied Biosystems, ThermoFisher Scientific) |
| Cq or raw data submission using RDML | **D** | Data availabe according to data availabilty statement. |

**References**

1. Tiwari, A. *et al.* Detection and quantification of SARS-CoV-2 RNA in wastewater influent in relation to reported COVID-19 incidence in Finland. *Water Res.* **215**, 118220 (2022).

2. Lu, X. *et al.* US CDC Real-Time Reverse Transcription PCR Panel for Detection of Severe Acute Respiratory Syndrome Coronavirus 2. *Emerg. Infect. Dis.* **26**, 1654–1665 (2020).

3. Pintó, R. M., Costafreda, M. I. & Bosch, A. Risk Assessment in Shellfish-Borne Outbreaks of Hepatitis A. *Appl. Environ. Microbiol.* **75**, 7350–7355 (2009).

4. Medema, G., Heijnen, L., Elsinga, G., Italiaander, R. & Brouwer, A. Presence of SARS-Coronavirus-2 RNA in Sewage and Correlation with Reported COVID-19 Prevalence in the Early Stage of the Epidemic in The Netherlands. *Environ. Sci. Technol. Lett.* **7**, 511–516 (2020).

5. ISO 15216-1:2017. Microbiology of the food chain, Horizontal method for determination of hepatitis A virus and norovirus using real-time RT-PCR, Part 1: Method for quantification. (2017).
